# Metasomatism and the crystallization of zircon megacrysts in Archaean peridotites from the Lewisian complex, NW Scotland

**DOI:** 10.1007/s00410-018-1527-5

**Published:** 2018-11-17

**Authors:** John W. Faithfull, Tim J. Dempster, John M. MacDonald, Monica Reilly, EIMF

**Affiliations:** 1 The Hunterian, University of Glasgow, Glasgow, G12 8QQ UK; 2 School of Geographical and Earth Sciences, University of Glasgow, Glasgow, G12 8QQ UK; 3 EIMF (Edinburgh Ion Microprobe Facility), School of GeoSciences, Edinburgh University, Edinburgh, EH9 3FE UK

**Keywords:** Zircon, Metasomatism, Megacrysts, Peridotite, Crustal melts, Lewisian

## Abstract

Zircon megacrysts are locally abundant in 1–40 cm-thick orthopyroxenite veins within peridotite host rocks in the Archaean Lewisian gneiss complex from NW Scotland. The veins formed by metasomatic interaction between the ultramafic host and Si-rich melts are derived from partial melting of the adjacent granulite-facies orthogneisses. The interaction produced abundant orthopyroxene and, within the thicker veins, phlogopite, pargasite and feldspathic bearing assemblages. Two generations of zircon are present with up to 1 cm megacrystic zircon and a later smaller equant population located around the megacryst margins. Patterns of zoning, rare earth element abundance and oxygen isotopic compositions indicate that the megacrysts crystallized from crustal melts, whereas the equant zircon represents new neocryst growth and partial replacement of the megacryst zircon within the ultramafic host. Both zircon types have U–Pb ages of ca. 2464 Ma, broadly contemporaneous with granulite-facies events in the adjacent gneisses. Zircon megacrysts locally form > 10% of the assemblage and may be associated to zones of localized nucleation or physically concentrated during movement of the siliceous melts. Their unusual size is linked to the suppression of zircon nucleation and increased Zr solubility in the Si-undersaturated melts. The metasomatism between crustal melts and peridotite may represent an analog for processes in the mantle wedge above subducting slabs. As such, the crystallization of abundant zircon in ultramafic host rocks has implications for geochemistry of melts generated in the mantle and the widely reported depletion of high field strength elements in arc magmas.

## Introduction

**Fig. 1:**
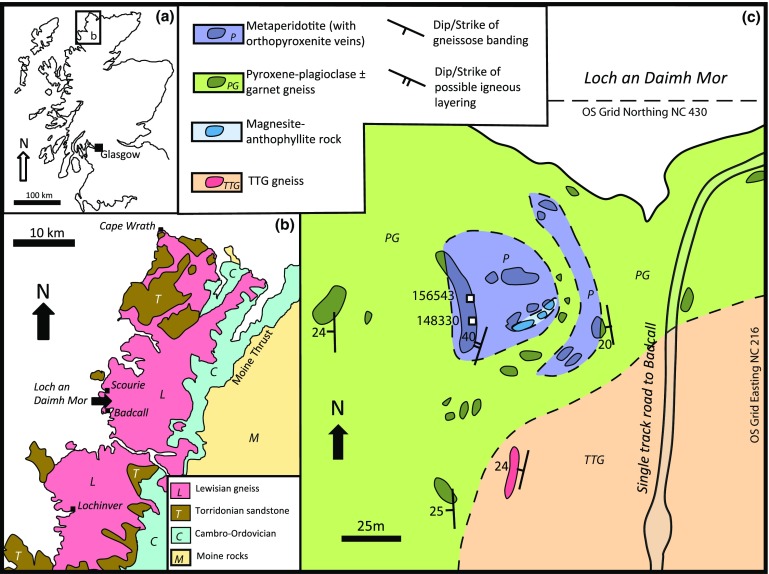
Maps showing. **a** Location of field area within NW Scotland. **b** Regional geology; and **c** geological map of area around the Loch an Daimh Mor metaperidotite. White squares show the two sample locations

Zircon is typically associated with crystallization from evolved igneous melts and the lack of zircon in mafic and ultramafic igneous rocks is commonly noted (e.g. Hoskin and Schaltegger 2003). However, zircon is frequently reported as xenocrysts in kimberlites (Belousova et al. 1998) and has been found in small quantities in peridotite from supposedly mantle wedge environments, as inherited grains from slab components (Liu et al. 2009; Li et al. 2016). Unfortunately such zircon often lacks the petrological context in which their generation in mantle peridotite might be assessed as they are either separated from their host rocks by natural processes or by sample preparation techniques. The Lewisian Gneiss Complex provides an excellent example of where the petrological context between zircon grains and ultramafic host rocks is preserved. Abundant coarse grained zircon up to 18 mm × 9 mm occurs in Archaean ultramafic rocks near Loch an Daimh Mor, just south of Scourie, NW Scotland (Fig. 1). Individual mechanically separated zircon from this locality has previously been isotopically dated using the U–Pb method and yield ages of 2,470 ± 30 Ma (Kinny and Friend 1997) and 2451 ± 14 Ma (Timms et al. 2006) and the microstructures within zircon have been described from this locality (Timms and Reddy 2009). However, there has been no petrological description of the unusual occurrence. The link between large zircon and ultramafic rocks is striking both here and at other localities in the Lewisian rocks of Scotland (e.g. specimen from Iona in the Hunterian museum collections: GLAHM 148,080, and from Eilean Glas, Scalpay National Museums of Scotland: Dudgeon collection 74-49-245 and 246). Similar occurrences of large zircon in ultramafic rocks have also been noted elsewhere, including Greenland (Nilsson et al. 2010), Italy (Marocchi et al. 2009), and from kimberlites (Kresten et al. 1975; Belousova et al. 1998; Robles-Cruz et al. 2012). However, the relationship of zircon to the ultramafic host is often uncertain.

In this study, we investigate this in situ mineral assemblage at Loch an Daimh Mor and present new field, petrographic, microtextural, geochemical and isotopic evidence on these exceptionally large and abundant zircon crystals and describe their petrological association with the Lewisian ultramafic host rocks. We suggest that these rocks record a common process involving metasomatic interaction between crustal melts and ultramafic host rocks with potentially important implications for the geochemical behaviour of high field strength elements (HFSE) in the mantle.

### Geological setting and previous work

The Lewisian Gneiss Complex (LGC) is dominantly composed of orthogneisses with tonalite–trondhjemite–granodiorite (TTG) protoliths, with subordinate mafic/ultramafic and metasedimentary gneisses (Peach et al. 1907). A number of discrete terranes are thought to be present within the gneiss complex with distinctive histories determined from structural and metamorphic field relationships and isotope geochronology (Kinny et al. 2005). The area around Loch an Daimh Mor is part of the Assynt Terrane, where zircon U–Pb ages range from ca. 3 Ga to 2.45 Ga, and have been variously ascribed to the formation of the igneous protolith, or to an early Badcallian metamorphic event, and/or a later Inverian event (Evans 1965; Park 1970; Corfu et al. 1994; Friend and Kinny 1995; Crowley et al. 2015). However, the difficulty in resolving and correlating the Badcallian and Inverian events in this polymetamorphic lower crustal unit is something that has plagued studies of the LGC since the inception of isotopic dating techniques (Kinny et al. 2005; Whitehouse and Kemp 2010). This confusion persists to this day in that granulite-facies metamorphism in the Assynt Terrane is commonly ascribed to the Badcallian event, but isotopic constraints also provide evidence of correlation with Inverian “age” events (Kinny et al. 2005; MacDonald et al. 2015). Despite improved analytical precision associated with zircon U–Pb ages, it seems that a spectrum of crystallization and/or Pb diffusion re-equilibration ages are produced from these high-grade gneisses (MacDonald et al. 2015) with a spread of 10 s to perhaps 100 s of millions of years. It is uncertain how much this range reflects discrete separate events, or just a prolonged, if perhaps episodic, high-temperature history.

The Archaean protoliths to the gneisses around Loch an Daimh Mor have experienced a granulite-facies tectonothermal event at 2482 ± 6 Ma (MacDonald et al. 2015), including partial melting (Johnson et al. 2012; Rollinson 2012), and locally, later amphibolite-facies event(s). Conditions during granulite-facies metamorphism nearby at Scourie are estimated to be in excess of 875 °C and ca. 9–11 kb (Johnson and White 2011; Cartwright and Barnicoat 1987). There is some evidence that granulite-facies event(s) may have been locally preceded by eclogite-facies metamorphism, with omphacite-bearing assemblages surviving in garnet-rich clots within some metagabbros (Sajeev et al. 2013), but the regional extent of this remains unclear. Later, at ca. 2400 Ma, the mafic Scourie Dyke Swarm was intruded (Heaman and Tarney 1989; Davies and Heaman 2014) post-dating the local granulite-facies events. The final episodes of tectonothermal activity occurred in Laxfordian events at ca. 1740 and 1670 Ma (Goodenough et al. 2013), characterized by shear zones and amphibolite facies retrogression.

The ultramafic bodies at Loch an Daimh Mor (Fig. 1b) occur ca. 2 km to the south of Scourie and were described by Peach et al. (1907), Bowes et al. (1964), and O’Hara (1961, 1965). They are surrounded by coarse grained granulite-facies garnet–clinopyroxene–plagioclase ± orthopyroxene mafic gneisses (Fig. 1c), probably metagabbros, and two-pyroxene tonalitic and mafic-banded orthogneisses (Rollinson and Windley 1980) with occasional metasedimentary xenoliths (Davies 1974). The ultrabasic bodies and adjacent mafic gneisses are considered to have experienced the same metamorphic grade (O’Hara 1961; Bowes et al. 1964; Sills et al. 1982). However, due to their different mineralogy, it is difficult to match metamorphic responses in the regional TTG gneisses to responses within the peridotite. The relative ages of the ultramafic bodies and the surrounding TTG gneisses are ambiguous. Many, especially the pyroxenite-dominated bodies, are probably later intrusive sills (Guice et al. 2018), but others (possibly including Loch an Daimh Mor) may be older than the host gneisses. For example, at “First Inlet”, just north of Scourie, a small ultramafic body is cut by a trondhjemite vein with 2955 Ma protolith zircon ages, similar to ages from the host TTG gneisses (Friend and Kinny 1995).

The presence of large zircon at Loch an Daimh Mor was first noted by mineral collectors such as Gordon Sutherland and Kemp Meikle in the 1960s. Specimens, from this time, usually labelled “Badcall”, are not uncommon in museum and private mineral collections in Scotland. The first published work on the zircon was by Kinny and Friend (1997), whose sample GST15 is described as “a large zircon 12.5 mm long hosted by pyroxenite from the margin of one of a series of ultrabasic/mafic bodies at Loch an Daimh Mor”. This sample has since been the subject of a range of other investigations including the effects of crystal plastic deformation of the large subhedral zircon (Timms and Reddy 2009) and U–Pb geochronology which yielded an age of 2,451 ± 14 Ma (Timms et al. 2006). Timms and Reddy (2009) stated that the deformation of the “syntectonic pyroxenite intrusion” hosting sample GST15 was associated with regional amphibolite-facies Inverian metamorphism. A brief note from a mineral collecting perspective has been published by Moffat and Starkey (2013). We present here the first detailed geological account of this ultramafic-hosted zircon occurrence.

### Field observations

#### The Loch an Daimh Mor peridotite

The peridotite occupies a total area of ca. 2500 m^2^ (Fig. 1c) and this study reports on relationships around UK grid reference NC1591 4291. Although the level of exposure is such that the geometry of the peridotite is uncertain, two sheet-like bodies are thought to be present aligned roughly parallel to the gently inclined foliation in the surrounding gneisses (Fig. 1c). A thin lower sheet of ca. 5 m thickness and an upper sheet of ca. 10 m thickness occur within garnet-rich pyroxene gneisses. Locally, concentrations of spinel occur within the peridotite with individual grains/clusters up to 1 cm. These may represent original igneous layering, which generally seems to be sub-parallel to the dominant regional fabric in the host gneisses (Fig. 1c). Only the lower boundary of the lower peridotite body is clearly exposed: serpentinized peridotite is interbanded on a cm-scale with garnet-rich pyroxene gneisses with a similar dip. The peridotite is typically close to dunite, with unfoliated, coarsely granoblastic olivine around 1–2 mm in diameter.

Partial alteration of olivine to serpentine is ubiquitous, with slickensided surfaces on fractures. An area dominated by anthophyllite–magnesiteanthophyllite–magnesite–talc assemblages (O’Hara 1965) occurs within the upper peridotite body (Fig. 1c), with coarse 0.5 cm randomly oriented anthophyllite needles present in 0.5 m diameter patches between 10 cm thick carbonate-rich veins. The anthophyllite–magnesite rock represents pervasively altered peridotite, retrogressed in lower amphibolite facies conditions, with high H_2_O and CO_2_ activity (Ford and Skippen 1997). Locally, sub-parallel 10 cm veins of this carbonated material cut serpentinized peridotite with sharp contacts. No low-temperature hydrous minerals equivalent to the anthophyllite-, or serpentine-bearing assemblages in the peridotite have been identified in the adjacent host gneisses. The only obvious retrograde effects are rare < 10 cm green amphibole-filled fractures in granulite-facies gneisses. Although the TTG and mafic gneisses are inherently less reactive than the peridotite to retrograde metamorphism, it is uncertain how the peridotite assemblages might relate to events in surrounding gneisses, or indeed how the required fluxes of H_2_O and CO_2_ entered the peridotite through those gneisses.

#### Veins in the Loch an Daimh Mor peridotite

**Fig. 2:**
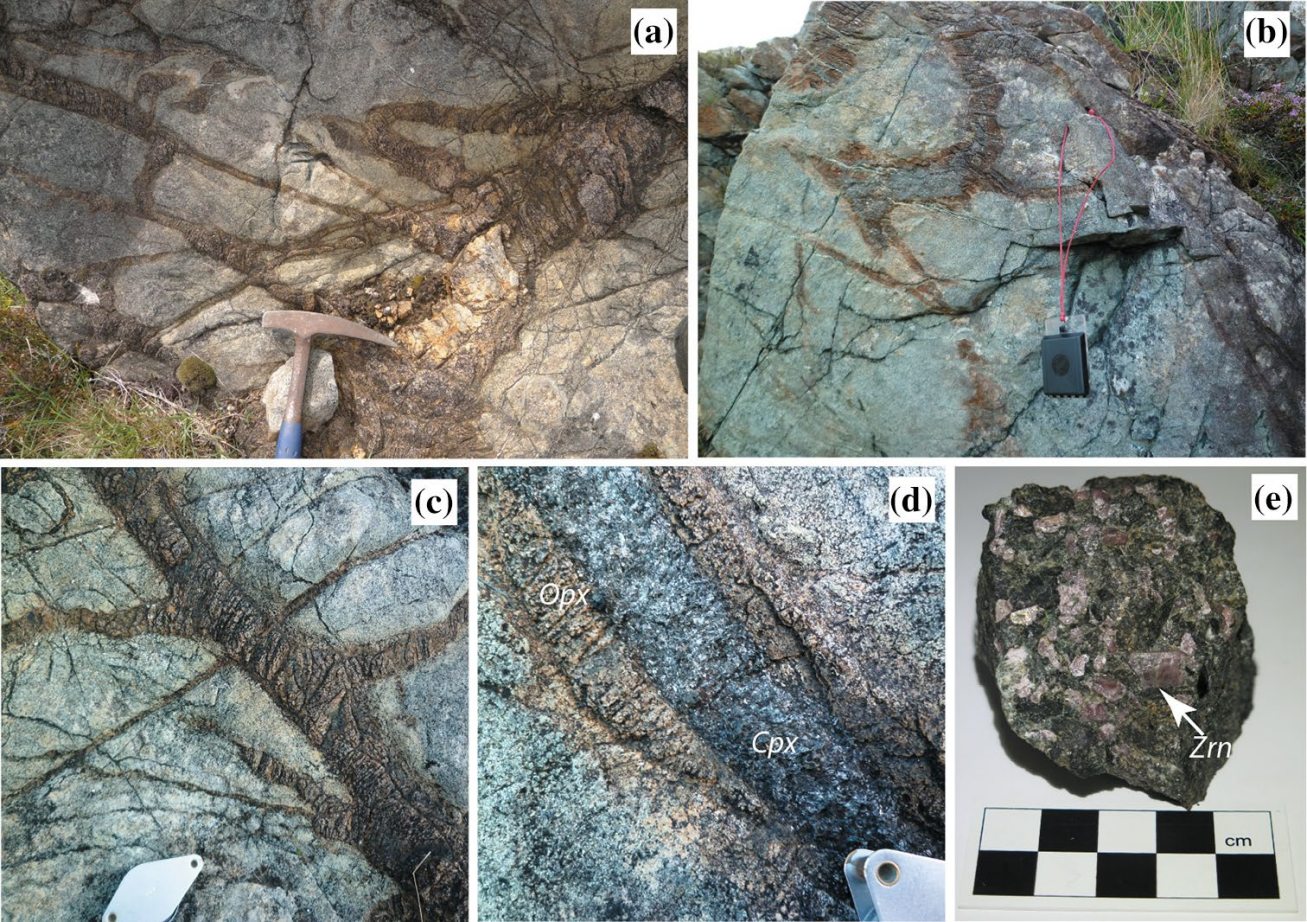
Field photographs of orthopyroxenite veins within the host peridotite. **a** Network of brown–orange orthopyroxenite. Hammer head is 20 cm long. **b** Irregular orthopyroxenite vein with central green clinopyroxene. Compass clinometer is 10 cm long; **c** Orthopyroxenite veins showing cross cutting relationships. Hand lens is 5 cm long; **d** Symmetrically zoned pyroxenite vein with central clinopyroxene (Cpx) and phlogopite zone and marginal orthopyroxene (Opx); **e** Hand specimen of serpentinized orthopyroxenite with several large pink zircon (Zrn) megacrysts

**Fig. 3:**
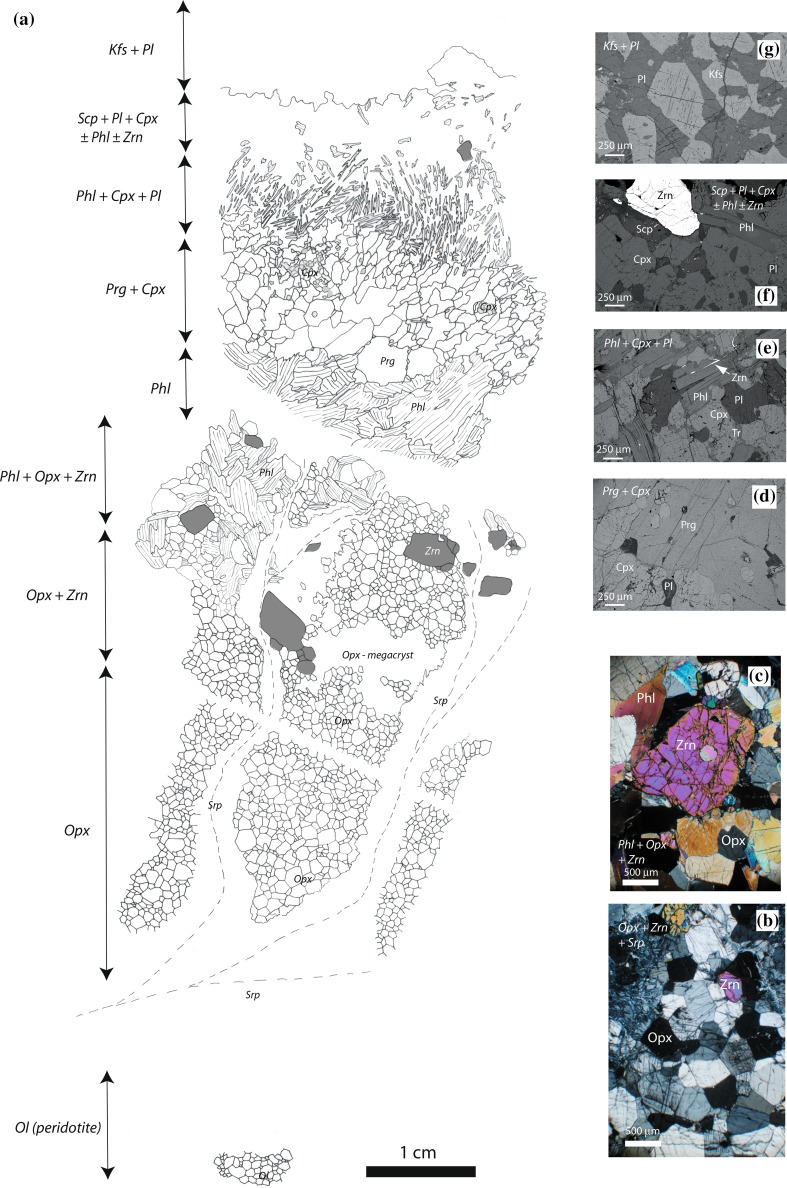
**a** Composite sketch showing petrography of thin sections from zoned orthopyroxenite vein with locations of zircon (Zrn—shaded) shown relative to different mineral assemblage zones labelled on left-hand side of the figure. Later veins of serpentinite (Srp) are common within both the host peridotite and the orthopyroxene veins. Transitions between some zones are absent due to either sample loss during thin section preparation or extensive serpentinization. Mineral abbreviations follow Whitney and Evans (2010). Each of the associated figures **b**–**g** is labelled in italics with the corresponding mineral zone of the zoned vein. **b** Photomicrograph of zircon megacryst within granoblastic orthopyroxenite (crossed polarized light); **c** photomicrograph of large zircon megacryst in phlogopite–orthopyroxene zone (crossed polarized light); **d** backscattered electron image of pargasite–clinopyroxene zone with minor altered interstitial plagioclase, and inclusions of pyroxene within pargasite. Clinopyroxene shows marginal alteration to tremolite–actinolite; **e** backscattered electron image of phlogopite–clinopyroxene–plagioclase zone with aligned phlogopite. Marginal alteration of pyroxene to tremolite–actinolite (Tr). Thin zircon locally occupies the cleavage of phlogopite (arrowed); **f** backscattered electron image showing isolated large zircon within scapolite–plagioclase–clinopyroxene zone; **g** backscattered electron image of leucomonzonite central part of the zoned vein with granoblastic plagioclase and perthitic orthoclase

Orange–brown cross cutting orthopyroxenite veins are the dominant structures present within the peridotite (Fig. 2). The veins are also partially altered to serpentine along both fractures and grain boundaries. The veins are common throughout the peridotite body, although present in greatest abundance (> 20%) in parts of the larger upper body. The orthopyroxenite veins range in thickness from ca. 1 cm up to 40 cm. They locally form networks (Fig. 2a, c), and typically occur as roughly planar sheets that can be traced over several metres. However, irregular-shaped veins are also present (Fig. 2b). Many veins dip at shallow to moderate angles to the northwest, but they are also in a range of other orientations forming sheets between blocks of the host peridotite. Some have an apparently folded geometry whilst others are displaced across minor fractures. Locally, veins cross-cut (Fig. 2c) but no consistent sequence of emplacement linked to orientation is identified. Larger volumes of orthopyroxenite are typically developed at the junctions between vein sets and tend to have the least regular geometry (Fig. 2b). Contacts between the orthopyroxene veins and the host peridotite are typically sharp. Most of the veins contain largely unfoliated, coarse (ca. 500 µm) granular pale grey orthopyroxene, with a little sulphide and locally abundant zircon megacrysts. However many show late fractures of a locally consistent orientation associated with serpentine alteration. Locally up to 1 cm orthopyroxene megacrysts are present in the veins and show marginal recrystallization to the more typical granoblastic orthopyroxene (Fig. 3a).

A few veins show a complex symmetrically zoned assemblage involving a sequence, or more typically partial sequence, of low-variance mineral assemblages (Fig. 2b, d). The thicker veins have both the most complete set of assemblages and the thickest individual zones, although the thickness of individual zones varies between different veins. The zoned veins are characterized by an outer margin (ca. 5 cm) of granoblastic orthopyroxenite (Fig. 3a, b), identical to that present in all of the veins. Typically the zoned veins contain a prominent central zone of dark green granoblastic clinopyroxene and pargasite (ca. 1.5 mm grains) with minor plagioclase that is a few cm thick but may be up to 20 cm (Fig. 2d). Clinopyroxene often appears as isolated inclusions within pargasite and as finer grained clusters surrounded by pargasite (Fig. 3d). The pargasite is both finer grained and shows shape alignment towards the more central parts of the vein. In some of the thicker veins, phlogopite (up to 5 mm) may be present in a thin cm-scale zone with orthopyroxene at the junction with an inner clinopyroxene/pargasite zone (Fig. 3a). The largest vein contains additional central plagioclase-bearing zones. An outer ca. 2 cm thick zone characterized by a symplectite-like intergrowth between aligned ca. 1 mm long euhedral phlogopite, with cleavage planes typically perpendicular to the vein margin, coarse grained (ca. 0.5 mm) clinopyroxene and plagioclase (Fig. 3a, e). Inside this, a zone with aligned phlogopite has a gradational contact with an inner ca. 1.5 cm thick zone where ca. 0.5 mm scapolite, clinopyroxene, plagioclase and less abundant phlogopite coexist (Fig. 3f). Trace amounts of apatite (up to 100 µm) and rutile are present typically within the phlogopite-rich zones. In this vein, there is a central core of leucomonzonite composed of coarse grained (0.5–1 mm) plagioclase and perthitic K-feldspar (Fig. 3a, g). Phlogopite typically shows patchy minor alteration to chlorite and calcite, and clinopyroxene may show patchy marginal retrogression to tremolite-actinolitic amphibole (Fig. 3e). Typically little alteration to serpentine occurs although some retrogression of original probable pyroxene is locally present in the zone with phlogopite–clinopyroxene–plagioclase intergrowths. The zoned structure does not extend along the full length of the vein but internal zones are laterally discontinuous and occur as isolated ellipsoids of variable length (Fig. 2b, d). This is most obvious with those veins containing just the additional clinopyroxenite central portion as they typically have the simplest geometry. Orthopyroxenite veins within the anthophyllite–magnesite rock body have an identical morphology to those in the adjacent peridotite, but appear to have been converted to pale fibrous amphibole-rich assemblages.

Large (mm to cm sized) pink zircon is visible in the field in several orthopyroxenite veins (Fig. 2e) within both of the peridotite bodies. Such megacrysts are difficult to observe on weathered surfaces, but are obvious on fresh surfaces. Zircon distribution was also assessed across field exposures at night using shortwave (254 nm) UV light as the mineral fluoresces bright yellow. Zircon is associated with either orthopyroxenite veins in the peridotite, or orthopyroxenite patches in deformed serpentinized peridotite (Fig. 3a–c). It is not present in the host peridotite and UV examination of the adjacent gneiss does not show large zircon megacrysts or abundant zircon. Coarse grained zircon is most abundant (up to 15% volume) in phlogopite-bearing orthopyroxenite but is also present in the pure orthopyroxenite typically close to the phlogopite–orthopyroxene zone (Fig. 3a, c). Zircon is occasionally present in the phlogopite-rich clinopyroxenite- and scapolite-bearing zones (Fig. 3f) but is absent from the central feldspathic zone. Typically the zircon megacrysts have a strongly clustered distribution within individual veins and lack obvious alignment (Fig. 2e). Isolated zircon rarely occurs within serpentinite, but always adjacent to orthopyroxenite. Most of the thinner orthopyroxenite veins do not contain obvious zircon, but where zircon occurs it is typically very abundant (Fig. 2e), and zircon-rich veins can be traced for several metres.

## Methods

Zircon morphology was characterized in polished thin sections by backscattered electron (BSE) and cathodoluminescence imaging using a FEI Quanta 200F field emission environmental scanning electron microscope operated at 20 kV. Composite BSE-reflected light-cathodoluminescence images that display the zoning and growth/alteration textures in the zircon were used to guide all subsequent chemical and isotopic analyses. Associated silicate minerals were analysed using the Carl Zeiss Sigma VP electron microscope SEM operated 20 kV, at the University of Glasgow. Major elements were measured by EDAX, using Oxford INCA software, and jadeite (Na,Al), rhodonite (Mn), garnet (Fe), diopside (Ca), periclase (Mg), chromite (Cr) and rutile (Ti) standards, with a 10-µm spot size to minimize sample heating effects. Nickel in olivine was measured using a WD detector, and Ni metal standard. Cl and S analysis of scapolite was carried out without standards using inbuilt calibration of INCA software.

The trace element and isotope geochemistry of the zircon was determined by SIMS analysis at the NERC Ion Microprobe Facility, University of Edinburgh. Trace elements were measured using a Cameca IMS-4F ion microprobe. Analytical and correction procedures follow those outlined by Kelly and Harley (2005) and Kelly et al. (2008). Analytical reproducibility of trace elements was calibrated against the 91,500 and SL1 zircon standard and the NIST SRM-610 glass standard (Hinton 1999). For most REEs (middle-heavy), the average analytical error was < 10% (2*σ*), but for some for the lighter REEs which have lower concentrations (La, Pr, Nd, Sm), it was higher. Analytical reproducibility against the NIST SRM610 glass standard was < 7% (2*σ*) for all analysed trace elements.

Oxygen isotope data were acquired at the University of Edinburgh with a Cameca IMS 1270 (#309), using a ~ 5 nA primary ^133^Cs^+^ beam. Secondary ions were extracted at 10 kV, and ^16^O^−^ (~ 3.0 × 10^9^ cps) and ^18^O^−^ (~ 4.0 × 10^6^ cps) were monitored simultaneously on dual Faraday cups (L’2 and H’2). Each analysis involved a pre-sputtering time of 60 s, followed by automatic secondary beam and entrance slit centring and finally data collection in two blocks of ten cycles, amounting to a total count time of 80 s, with an analysis diameter of ca 15 µm. The external precision of each analysis is < 0.2‰. To correct for instrumental mass fractionation (IMF), all data were normalized to a Zircon Standard 91,500 (Wiedenbeck et al. 2004) mounted together with the samples and measured throughout the analytical sessions. The internal precision is estimated from the repeat analysis of the standard to be < 0.17‰.

U–Pb isotopic determinations were also acquired using the Cameca IMS 1270 and follow methods described in Kelly et al. (2008) and ratios were calibrated against 91,500 zircon (Wiedenbeck et al. 2004). Reproducibility on standard measurements during the analysis was ca 1% (1*σ*). Analysis spot size was ca 25 × 30 μm. Concordia plots and age calculations follow Ludwig (2003). Common Pb corrections (typically < 10 ppb) were determined using ^204^Pb and potential surface contamination reduced by routinely discarding the first five sets in the analytical cycle.

Analysed samples (GLAHM 148,330, 156,543) and other representative material are deposited in the Hunterian, University of Glasgow.

## Results

### Zircon textures

**Fig. 4:**
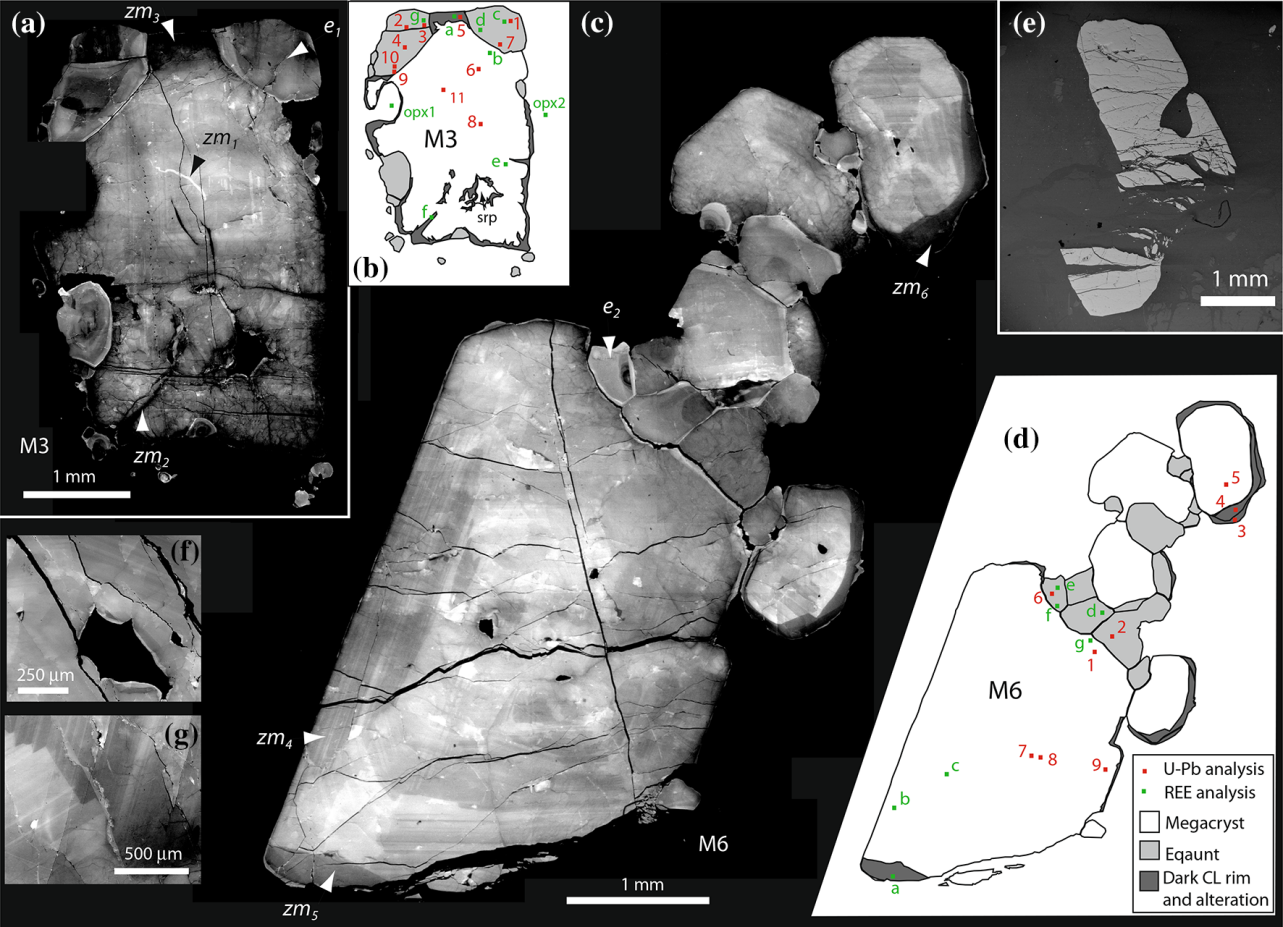
Cathodoluminescence images (unless otherwise stated) of zircon megacrysts. **a** Large megacryst (M3) with irregularly zoned and fractured interior, equant granular zircon forms partial inclusions around the top corners and lower left-hand side. zm_1_—oscillatory zoned megacryst core with bright CL fracture fill; zm_2_—dark CL fracture fill in irregularly fractured megacryst host; zm_3_—dark CL altered rim to megacryst; e_1_—zoned equant zircon with bright CL rim on inner margin. **b** Cartoon representation of zircon M3 showing main types of zircon and locations of U–Pb and REE analyses. **c** Large zircon megacryst (M6) with well-developed oscillatory zoning, marginal modification producing equant granular zircon. zm_4_—oscillatory zoned megacryst with irregular zoning in the core area; zm_5_—dark CL marginal alteration to large megacryst; zm_6_—dark CL overgrowth to small sector zoned megacryst zircon with fine-scale irregular fractures; e_2_—zoned equant zircon with bright CL rim. **d** Cartoon representation of zircon M6 showing main types of zircon and locations of U–Pb and REE analyses. **e** Fractured megacryst showing displacement along serpentine-filled fractures (Backscattered electron image). **f** Zoned, fractured zircon showing bright CL modification around phlogopite inclusion (dark CL). **g** Oscillatory zoned zircon with bright, altered zircon showing crystallographic control on the modification either side of the central fracture

Two main generations of zircon are present within the orthopyroxenite veins: larger megacrysts and smaller equant zircon. Large cm-sized subhedral to euhedral zircon megacrysts (Fig. 4a, c) are abundant in some veins with locally well-developed concentric fine-scale (1–100 µm) oscillatory zoning (Fig. 4c). This zoning in cathodoluminescence is typically most well developed in the thick (up to 1 mm) outer mantles of the megacrysts around an apparently more uniform core. All boundaries between core and mantle zones are approximately parallel to the megacryst edge. Sector zoning is present in some grains typically with brighter luminescence zones radiating towards the corners (Fig. 4c, g). Outer zones of the megacrysts contain broader zones locally with dark luminescent outer rim of between 30 and 400 μm thickness (Fig. 4a, c). This rim may either have a sharp, locally cross-cutting, contact with the internal zones or there may be a continuous transition into the brighter internal zones. Locally the zircon megacrysts contain a thin (ca. 20 μm) discontinuous more brightly luminescent zone on the outer edge (Fig. 4c), either when in contact with other silicates or in contact with other zircon. The dark CL outer rims are cut across by both the equant zircon and the local outer bright CL rims. Inclusions of orthopyroxene and more commonly phlogopite, up to 500 μm, are present, which may alter to serpentine in some instances. Smaller inclusions, both mineral and probably fluid, may form linear arrays within the zircon host associated with fractures filled by more strongly luminescent zircon. The zircon megacrysts are characterized by several generations of fractures (Fig. 4a, c, e, f), also noted in the studies of Timms et al. (2006), Rimsa et al. (2007) and Reddy et al. (2006). Cathodoluminescence images show that the large zircon is commonly pervasively fractured, typically with no or very minor shear displacement, although some larger serpentine-filled fractures are associated with brecciation and displacements up to 1 mm (Fig. 4e). A variety of different fracture types are present: pervasive, branching fractures of random orientation characterized by dark luminescent zircon of a few microns to tens of microns width (Fig. 4a); fractures with more consistent orientation filled by bright luminescent zircon (Fig. 4a); and sets of concentric fractures centred on points at the edges of some zircon. The intense fracturing may create a blocky style of zoning in the cores of some large megacrysts, with broad fracture-related zones of dark luminescent zircon superimposed on areas with oscillatory zoning (Fig. 4a). Bright luminescent zircon-filled fractures may be in continuity with the thin zones of bright luminescent zircon forming at the very edge of the large zircon. Such brightly luminescent zircon may also form a thin (ca. 20–50 µm) edge of the zircon adjacent to the larger inclusions (Fig. 4f) and is often associated with an adjacent dark inner zone of similar width. Some fractures associated with bright luminescent zircon may follow consistent crystallographic orientations within the zircon and show an asymmetric pattern of bright luminescent zircon either side of the plane (Fig. 4g).

**Fig. 5:**
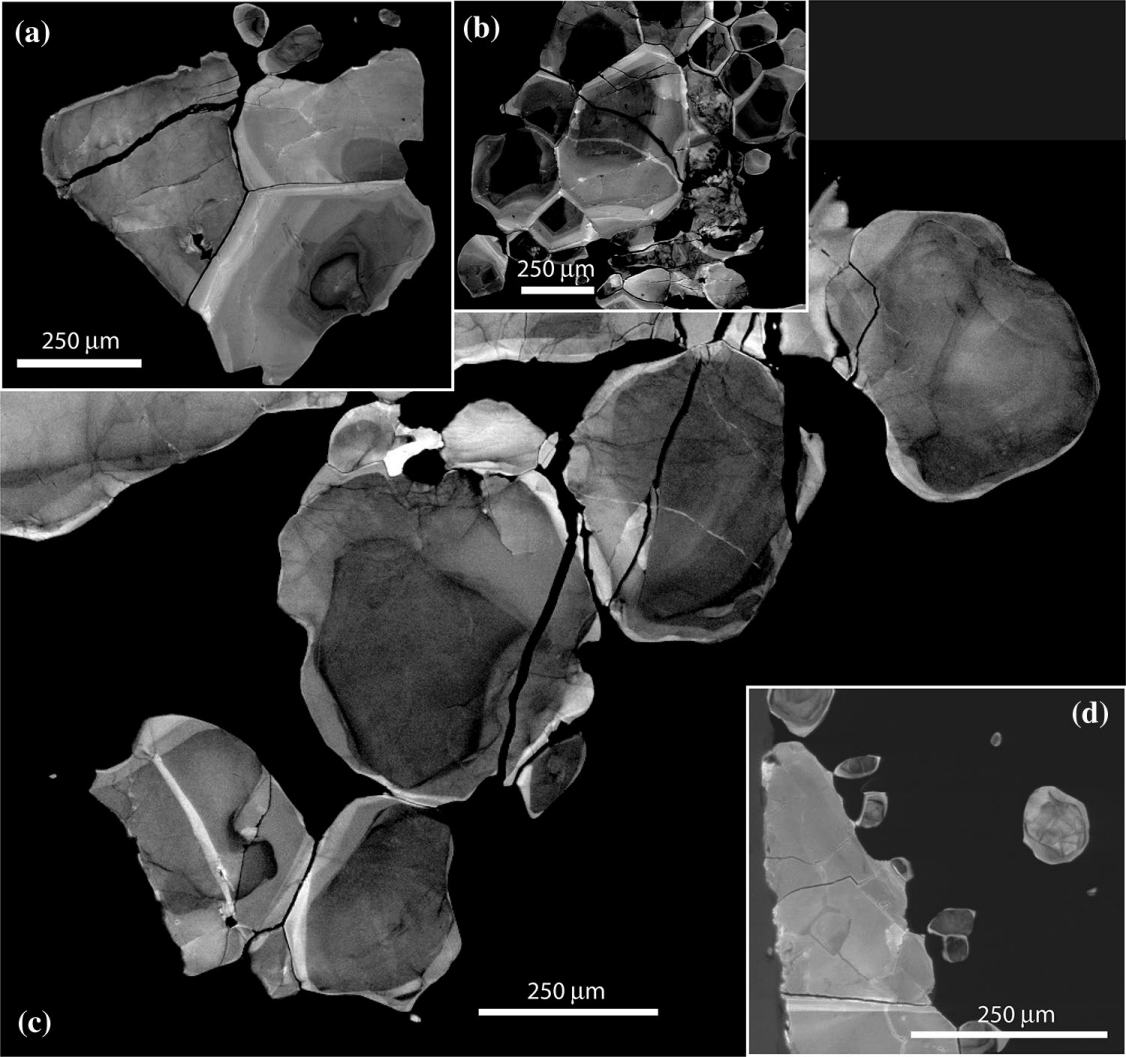
Cathodoluminescence images of equant granular zircon. **a** Two large equant zircons adjacent to fragment of fractured megacryst zircon (left side of image). **b** Cluster of zoned equant zircon with granoblastic texture and both concentric and asymmetric zoning. **c** Zoned granular zircon containing a range of different fracture types with both dark and light CL fill. **d** Megacryst zircon with smaller equant zircon present around right hand margin, showing thin isthmus of late zircon growth joining the equant and megacryst zircon

A generation of smaller (100–500 µm) equant zircon form clusters or aligned trails around margins of larger megacrysts (Fig. 5). These have a granoblastic texture within each cluster (Fig. 5b) and also commonly occur as individual partially enclosed inclusions within the outer margins of the larger grains (Fig. 4a). Some of these granular inclusions are located in consistent crystallographic positions at the corners of the megacrysts but there is no obvious relationship between the crystallographic orientation of the megacrysts and that of the later granular zircon. The equant zircon that occurs within the edge of the megacrysts are typically larger than those occurring in the adjacent clusters. Locally the small equant zircon appears to be evenly spaced along the edge of the large zircon and the edge of the latter protrudes towards the former (Fig. 5d), forming a partial or complete isthmus between the two. The equant zircon is characterized by rather darker luminescence than the megacrysts, and has broad (50–100 µm) diffuse concentric zones typically with a darker luminescent core and brighter rim (Fig. 5a–c). The zones are not sharply concentric but are typically smoothly curving broadly parallel to the grain boundaries (Fig. 5a). Isolated equant zircon lacking contact with other zircon typically shows concentric zoning geometry (Fig. 5d), whereas those adjacent to other zircon, whether megacrystic or granular types, typically show asymmetric zoning, with a geometry either indicative of growth out from a granoblastic cluster (Fig. 5c) or growth towards the megacrystic “host” grain (Fig. 5a). As with the megacryst zircon, edges of the equant zircon are commonly characterized by a bright rim that is present between the inclusions and the host grain and between the smaller grains in clusters around the megacrysts (Fig. 5c). The smaller equant zircon is characterized by less fracturing, especially those fractures filled by the darker CL zircon. Some serpentine-filled fractures are present and those filled with bright luminescence zircon may be in continuity with the local bright luminescent rims (Fig. 5c).

The phlogopite-rich zones of the orthopyroxenite vein contain a rare third form of zircon, which was not analysed. This “skeletal” thin (20 µm) elongate (300 µm) zircon occurs between [001] cleavage planes of phlogopite and extends along adjacent grain boundaries (Fig. 3e).

### Mineral chemistry

The major element compositions of minerals from the host peridotite and the orthopyroxenite vein assemblage are presented in Table 1. Olivine in the peridotite has a composition Fo_87_, and those other ferro-magnesian mineral phases in the vein assemblage are similarly Mg-rich. Hence, the orthopyroxene has Mg # of 86.5–87.6, with an Al content of 2.6–3.6% Al_2_O_3_. Generally, the Mg numbers of both clinopyroxene (and phlogopite (88 − 84) decrease slightly towards the centre of the zoned veins (Table 1) and plagioclase is progressively more sodic (An_55_ to An_35_) within the more central zones (Table 1). Most phases themselves are chemically unzoned in the veins, although minor marginal retrogression of the diopside-rich clinopyroxene occurs, producing a Mg-rich hornblende. Rare spinels within the orthopyroxenite have a Cr/(Cr + Al) of 0.22. Scapolite is relatively Ca-rich with a Ca/(Ca + Na) of 0.73, with SO_3_ up to ca 7% (cf. Teertstra et al. 1999).

**Table 1:** Representative major element analyses (wt%) of minerals from different zones in the veins

Mineral	Zone	SiO_2_	TiO_2_	Al_2_O_3_	FeO	MnO	MgO	CaO	Na_2_O	K_2_O	Cr_2_O_3_	NiO	Total	Mg #	SO_3_	Cl	O=Cl	Total
ol*	ol	40.57	bd	0.10	12.01	0.12	47.56	bd	0.09	bd	bd	0.38	100.82	88				
ol*	ol	39.52	bd	0.16	12.00	0.16	46.54	bd	0.08	bd	0.08	0.44	98.97	87				
opx	opx	54.36	0.13	2.92	8.85	0.22	31.95	0.37	bd	bd	0.35	0.07	99.22	87				
opx	opx	55.46	0.10	2.94	8.74	0.15	32.31	0.20	0.06	bd	0.24	0.05	100.23	87				
cpx	opx	52.73	0.47	3.15	3.33	bd	16.69	22.90	0.40	bd	0.30	0.02	99.99	90				
spl	opx	0.34	bd	41.78	22.56	0.24	13.23	0.05	0.55	bd	17.89	0.36	97.00	51				
phl	phl	38.64	2.49	15.26	5.42	bd	21.29	0.08	0.50	9.40	0.13	0.14	93.34	88				
cpx	phl	52.65	0.18	4.12	4.00	0.11	15.13	23.02	0.70	bd	0.11		100.01	87				
amp*	phl	54.65	0.25	4.11	3.73	bd	21.34	12.93	0.70	0.15	0.18	0.17	98.22	91				
amp	prg	42.79	1.23	14.12	6.22	0.06	15.86	11.99	1.83	1.82	0.20	0.01	96.12	82				
amp	prg	43.00	1.28	14.33	6.52	0.10	16.07	12.14	1.68	1.87	0.31	0.13	97.43	82				
phl	prg	38.63	2.56	15.37	5.95	bd	21.33	0.05	0.51	9.74	0.10	0.11	94.34	87				
cpx	prg	52.66	0.15	3.75	4.58	0.09	15.34	22.23	0.79	bd	0.07		99.66	86				
cpx	prg	52.23	0.07	3.36	4.32	0.13	15.06	22.91	0.63	bd	0.11	0.04	98.87	86				
pl	prg	53.70	bd	28.50	0.06	bd	0.20	11.17	5.03	0.11	bd		98.77					
cpx	phl-cpx-pl	52.15	0.08	3.48	4.38	0.15	15.20	22.91	0.69	bd	0.16	0.01	99.22	86				
pl	phl-cpx-pl	53.86	0.08	28.48	0.09	bd	0.11	11.13	5.25	0.05	bd		99.05					
amp*	phl-cpx-pl	49.31	0.35	7.92	5.70	0.12	18.22	12.49	0.98	0.57	0.11	0.01	95.78	85				
amp	phl-cpx-pl	43.11	0.97	14.31	7.44	0.08	15.24	12.09	1.80	1.54	0.05	0.10	96.73	79				
phl	phl-cpx-pl	39.73	2.08	15.20	6.01	bd	20.83	0.19	0.08	9.66	bd	0.27	94.04	86				
cpx	int-scp	53.12	0.12	2.97	3.74	0.14	15.79	23.24	0.71	bd	bd		99.83	88				
scp	int-scp	45.80	bd	26.82	0.20	bd	0.64	17.30	3.53	0.07	0.08	0.01	94.45		5.79	0.31	0.07	100.48
pl	int-scp	54.59	bd	28.49	0.18	bd	0.09	10.81	5.42	0.07	bd	0.01	99.67					
phl	int-scp	38.99	2.03	15.26	6.88	bd	20.86	0.06	0.14	10.10	0.06	0.10	94.49	85				
amp*	int-scp	55.85	bd	3.48	4.52	0.10	21.68	12.71	0.42	0.21	0.30	0.02	99.30	90				
scp	scp	45.73	bd	26.49	0.26	bd	0.59	17.30	3.40	0.06	0.12		93.96		6.66	0.22	0.05	100.79
scp	scp	45.77	bd	26.44	0.24	bd	0.65	17.31	3.55	0.09	0.10	0.04	94.19		3.92	1.57	0.35	99.33
pl	scp	54.87	0.22	28.46	0.17	bd	bd	10.91	5.88	0.23	bd		100.75					
pl	scp	53.77	bd	28.17	bd	bd	0.05	10.86	5.19	bd	bd	0.05	98.08					
cpx	scp	53.13	0.18	2.80	4.11	0.10	15.42	23.27	0.68	bd	0.10		99.79	87				
phl	scp	39.39	1.95	15.26	6.96	0.08	20.96	bd	0.22	9.77	0.06		94.66	84				
cpx	kfs-pl	53.51	0.11	2.00	4.34	0.13	15.55	23.38	0.58	bd	0.07	0.02	99.69	87				
pl*	kfs-pl	57.75	0.05	27.76	0.05	bd	0.09	9.49	6.50	0.16	0.07		101.92					
pl	kfs-pl	58.85	bd	25.66	0.05	bd	0.11	7.63	7.27	0.09	bd	0.01	99.68					
pl	kfs-pl	59.49	bd	25.68	bd	bd	0.07	7.34	7.58	0.20	bd	0.04	100.40					

Mineral abbreviations follow Whitney & Evans (2010). EDS concentrations < 0.05 wt% have been treated as below detection (bd) limits

ol* host peridotite, *Olivine* spinel opx points analysed on Specimen GLAHM 148,330, other zones on GLAHM 156,543, amp* secondary amp after cpx, pl* pl inclusion in cpx, *int-scp* zone with scapolite intergrowths

### Trace elements

The two main populations of zircon, megacrystic and equant types are characterized by relatively consistent and distinctive trace element geochemistry. All of the zircon from the orthopyroxenites have consistent Hf contents of 8617 ± 374 ppm (2*σ* error) (Table 2), irrespective of textural type.

**Table 2:** Trace element analyses (ppm) for zircons M3 and M6 (Fig. 4) and adjacent granoblastic orthopyroxenes

	M6a	M6b	M6c	M6d	M6e	M6f	M6g	M3a	M3b	M3c	M3d	M3e	M3f	M3g	Opx1	Opx2
	Mega	Mega	Mega	Equant	Equant	Equant	Equant	Mega	Mega	Equant	Mix	Mega	Mega	Mix		
Ca	13.31	13.48	15.34	15.13	20.61	16.79	34.63	10.46	10.41	26.62	33.08	18.72	26.75	14.92	1556.10	2651.60
Ti	6.53	8.04	9.84	6.98	15.42	12.49	5.82	8.23	9.92	8.58	8.71	10.98	12.19	9.03	410.48	484.83
Y	334.07	408.44	282.27	54.17	50.23	56.44	71.78	282.21	222.96	50.12	104.52	262.42	333.35	111.21	0.27	1.60
Nb	2.13	2.87	1.20	1.29	0.76	0.30	0.00	na	0.00	0.08	0.00	0.62	0.22	0.02	0.03	0.01
Zr	486,490	486,020	478,170	469,060	479,200	479,060	479,570	473,906	476,422	473,033	476,547	486,066	478,164	473,607	0.76	3.33
Ba	0.00	0.28	0.00	0.29	0.15	0.32	0.35	0.36	0.16	0.37	0.35	0.23	0.49	0.00	0.11	0.15
La	0.04	0.03	0.06	0.01	0.04	0.05	0.05	0.06	0.09	0.05	0.02	0.05	0.08	0.03	0.02	0.03
Ce	29.55	18.16	13.19	4.00	2.46	2.31	2.34	26.45	16.81	2.83	2.77	20.73	37.86	3.41	0.02	bd
Pr	0.12	0.11	0.26	0.02	0.02	0.03	0.02	0.15	0.29	0.01	0.02	0.23	0.30	0.02	0.01	0.01
Nd	1.65	1.93	2.15	0.37	0.08	0.15	0.16	2.05	2.63	0.32	0.33	1.90	3.63	0.50	bd	bd
Sm	2.03	2.38	2.09	0.38	0.19	0.13	0.23	2.27	1.69	0.14	0.09	1.53	2.58	0.38	bd	bd
Eu	0.27	0.48	0.36	0.15	0.07	0.11	0.11	0.45	0.31	0.06	0.11	0.26	0.34	0.06	bd	bd
Gd	7.42	9.19	7.29	0.97	0.71	0.59	0.92	6.34	6.17	1.09	0.97	5.83	9.07	1.38	bd	bd
Tb	2.15	3.13	2.04	0.22	0.20	0.26	0.34	1.97	1.44	0.22	0.37	1.67	2.34	0.37	0.02	0.02
Dy	24.78	31.82	24.73	2.92	2.26	3.11	4.10	22.34	17.65	3.01	5.14	19.57	23.94	6.77	bd	0.08
Ho	9.14	12.38	8.72	1.49	1.13	1.39	1.73	7.85	6.16	1.16	2.94	7.64	8.87	2.90	0.01	0.06
Er	45.45	55.22	37.47	5.87	5.23	7.22	8.68	41.26	29.55	5.41	14.29	35.04	44.42	14.86	0.06	0.40
Tm	9.23	10.62	7.71	1.24	1.36	1.64	2.15	7.82	5.97	1.14	3.88	7.03	9.25	3.58	0.03	0.06
Yb	73.31	83.81	61.84	10.39	11.27	15.61	20.19	63.04	45.27	11.15	34.31	56.41	72.56	38.18	0.30	0.21
Lu	17.99	20.46	14.11	2.49	3.66	4.50	5.32	15.88	12.56	3.19	10.27	14.05	17.78	10.19	0.05	0.12
Hf	9838	8431	8390	8490	8579	8436	8667	8602	8508	8481	8436	8829	8630	8325	0.07	0.22
Ta	72.42	74.21	73.51	69.05	68.91	68.46	72.39	na	na	na	na	na	na	na	0.07	bd
Th	32.89	20.20	14.65	33.39	19.44	13.72	8.30	48.25	17.08	18.91	11.50	17.91	61.99	9.81	bd	bd
U	44.46	25.19	21.10	59.01	25.53	22.90	15.61	40.03	21.17	23.45	18.33	21.87	49.42	17.01	bd	0.04

*mega* megacrystic zircon, *equant* equant or granular zircon, *mix* transitional chemistry, shading indicates dark CL zircon, *na* not analysed, *bd* below detection limits

**Fig. 6:**
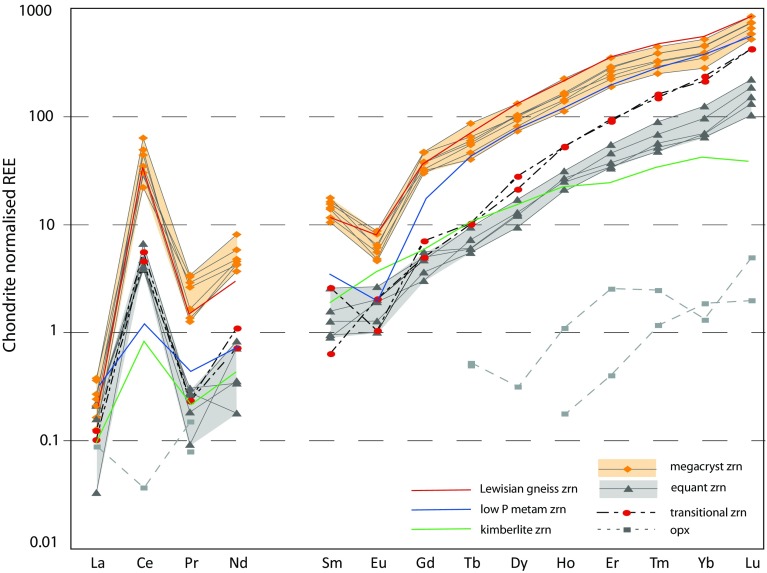
Chondrite-normalized REE plot, comparing megacryst zircon and equant zircon compositions. Brighter CL rims to the equant population have a transitional composition with HREE similar to the megacrysts and LREE similar to the equant zircon. Partial REE profiles of granoblastic orthopyroxene also shown, some LREE are below detection limits. Compositions of zircon from Lewisian mafic gneisses (MacDonald et al. 2015); low pressure metamorphic zircon (Rubatto and Hermann 2007) and zircon from depleted mantle source (Robles-Cruz et al. 2012) are shown for comparison

The zircon megacrysts have relatively high total REE contents (av. 201 ppm), high *Y* contents (408–223 ppm), positive Ce-anomalies (Ce/Ce* = 57.8), and negative Eu anomalies (Eu/Eu* = 0.28) (Fig. 6) (Table 2) in comparison to the equant zircon. The chondrite-normalized REE profiles of the megacrysts (Fig. 6) show characteristically high Sm/La ratios (65.9) and low Lu/Gd ratios (17.9). Ti contents of the zircon megacrysts range from 6.5 to 12.1 ppm. Luminescence intensity generally shows an inverse correlation with actinide content, but is unrelated to other trace elements. Dark luminescence zircon associated with fracturing and late alteration is characterized by typically 2–3 times higher U and Th contents with slightly higher Th/U (mean = 1.07) than the brighter CL zones of the megacrysts (mean Th/U = 0.78). As such the actinides appear to be the most mobile of the trace elements within zircon (Timms et al. 2006).

The smaller, equant zircon grains form a geochemically distinct population characterized by consistently low total REE contents (av. 34 ppm), and correspondingly low Y (50–72 ppm). They lack a marked Eu anomaly (Eu/Eu* = 0.74) (Table 2; Fig. 6) and have a slightly less prominent Ce anomaly (Ce/Ce* = 31.9) than the megacrysts. The equant zircon has low Sm/La (20.5) ratio and high Lu/Gd ratio (39.0) relative to the megacryst zircon. The brighter luminescent edges of the equant zircon, immediately adjacent to the megacrysts, may have a transitional REE chemistry (total REE = 79 ppm) between the typical granular zircon and the megacryst zircon that they replace. Thus, they have high HREE concentrations characteristic of the megacryst composition and lower LREE content (Lu/Gd = 72.6, Sm/La = 13.6) and have either a small negative Eu anomaly or lack a Eu anomaly (Fig. 6; Table 2). The latter features are characteristic of the equant zircon. The equant zircon has a similar range of Ti contents to the megacrysts (5.8–15.4 ppm). Actinide contents of the equant zircon are very similar to those of the megacrystic forms, with greater variation between dark and bright luminescent zircon within a single textural type than between different textural types.

### Oxygen isotopes

**Fig. 7:**
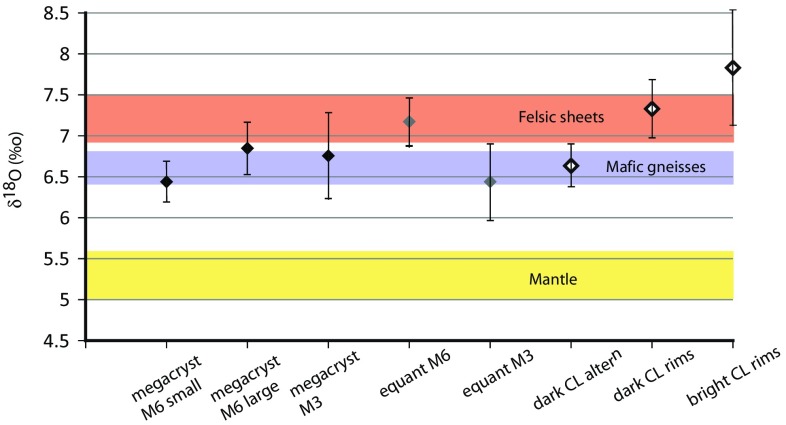
Average oxygen isotopic compositions of different textural types of zircon, with comparison to published analyses of zircon from mantle reservoirs from Valley et al. (1998) and Lewisian mafic gneisses and felsic melts (Cartwright and Valley 1992)

The oxygen isotopic values of the megacrystic (*δ*^18^O = 6.72 ± 0.37‰ 2*σ* error) and equant zircon (*δ*^18^O = 6.82 ± 0.55‰) are identical (Table 3; Fig. 7). Although the megacrysts do show some internal variation, it is not linked to any obvious spatial constraints or CL-defined zones. Different megacrysts appear to have identical average oxygen isotopic values. Equant zircon appears to show variation in isotopic composition with those spatially associated with the two individual megacrysts having possible slight differences in *δ*^18^O with 7.15 ± 0.29‰ and 6.44 ± 0.46‰ (Fig. 7). Although zircon rim compositions were difficult to analyse due to topographic variation at the edges of grains these yield possibly higher *δ*^18^O = 7.56 ± 0.64‰ values, irrespective of whether they have a bright or dark CL character. The megacrystic zircon that shows dark CL character typically adjacent to the rims of the grain (i.e. similar to the dark CL rims), has lower *δ*^18^O = 6.64 ± 0.25‰ effectively identical to the host megacryst composition (Fig. 7).

**Table 3:** Oxygen isotope data for megacrysts and equant zircon (M3 and M6, Fig. 4)

M6 large megacryst		M3 large megacryst	
Grain spot	*δ* ^18^O (‰)	Grain spot	^18^O (‰)
M6 m21	7.30	M3 m37	6.27
M6 m22b	7.84	M3 m42	6.70
M6 m23	7.07	M3 m43	7.31
M6 m24	6.56	M3 m36d	6.40
M6 m25	6.33		
M6 m28	6.88	M3 equant	
M6 m29	6.67	Grain spot	δ^18^O (‰)
M6 m30	7.10	M3 e31	6.56
		M3 e32b	7.07
M6 small megacrysts		M3 e33	6.26
Grain spot	δ^18^O (‰)	M3 e34	6.28
M6 ms2d	6.76	M3 e35b	6.43
M6 ms3d	6.46	M3 e38	6.23
M6 ms4	6.44	M3 e39	5.93
M6 ms5	6.76	M3 e40	7.39
M6 ms6	6.18	M3 e41b	8.74
M6 ms7d	7.02		
M6 ms8	6.37		
M6 ms9d	6.55		
M6 ms10do	7.57		
M6 ms11do	7.08		
M6 ms26do	6.13		
M6 ms27	6.85		
M6 equant			
Grain spot	δ^18^O (‰)		
M6 e12	6.87		
M6 e13	7.55		
M6 e14	7.10		
M6 e15	7.13		
M6 e16b	7.75		
M6 e17	7.37		
M6 e18	6.87		
M6 e19	7.59		
M6 e20	6.86		

Bright (b) CL rims

Dark (d) CL rims and overgrowths (o)

### U–Pb geochronology

**Fig. 8:**
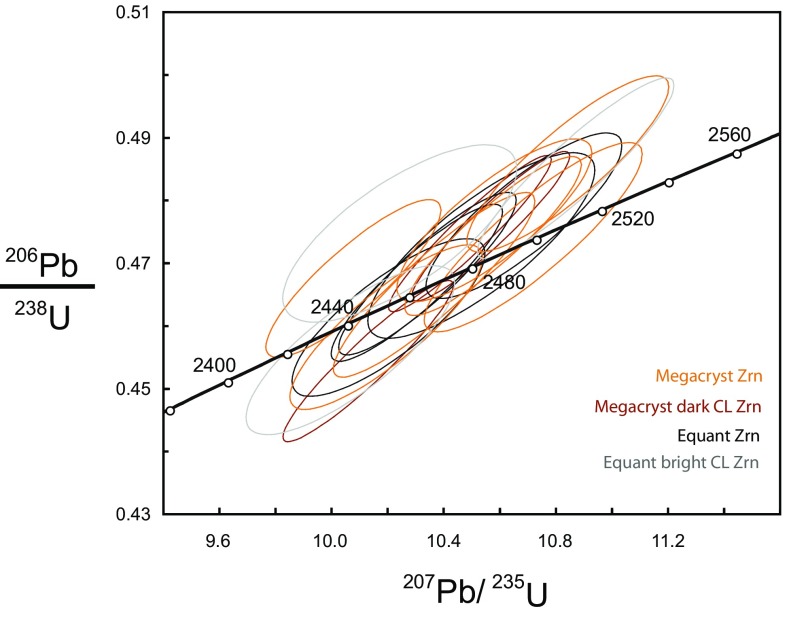
U–Pb concordia plot of both megacrystic and equant zircons with 2*σ* error ellipses.

Nearly all U–Pb isotopic analyses (Table 4) are within error of the concordia line (Fig. 8) with the whole population recording a weighted average ^207^Pb/^206^Pb age of 2464 ± 8 Ma (2*σ* error, *n* = 20, MSWD = 2.8). Hence, there is no significant Pb loss and the U–Pb ages are interpreted as dating crystallization of the zircon. The weighted average ^207^Pb/^206^Pb age of megacrysts is 2464 ± 12 Ma (2*σ* error, *n* = 11, MSWD = 3.8), this is within analytical error of the weighted average ^207^Pb/^206^Pb age of the equant zircon at 2465 ± 12 Ma (2*σ* error, *n* = 9, MSWD = 1.8) (Fig. 8). Although dark luminescent rim compositions are significantly enriched in both U and Th, there is no discernable difference in the isotopic ages of these rims. Two of the zircon analyses record younger ages, of these one (M3-8 m) has relatively high common Pb, the other (M3-7 eb) is slightly discordant and was obtained was from the very bright luminescent edge of the equant zircon with a ^207^Pb/^206^Pb age of 2418 ± 19 Ma.

**Table 4:** Summary of U/Pb zircon results for zircons M3 and M6 (Fig. 4)

	U (ppm)	Th (ppm)	Pb (ppm)	Th/U	f_206_ (%)	Isotopic ratios							*ρ*	Age (Ma)						D (%)
						^204^Pb/^206^Pb	^207^Pb/^235^U	1*σ*	^206^Pb/^238^U	1*σ*	^207^Pb/^206^Pb	1*σ*		^206^Pb/^238^U	1*σ*	^207^Pb/^235^U	1*σ*	^207^Pb/^206^Pb	1*σ*	
M6-1 m	33	29	19	0.91	0.29	0.00016	10.580	0.142	0.477	0.005	0.161	0.001	1.00	2513	23	2487	12	2465	13	2.0
M6-2 e	44	49	27	1.13	0.14	0.00007	10.342	0.129	0.468	0.005	0.160	0.001	1.01	2476	23	2466	12	2457	9	0.8
M6-3 mdo	142	110	81	0.80	0.10	0.00006	10.567	0.115	0.476	0.005	0.161	0.001	1.01	2509	21	2486	10	2466	7	1.8
M6-4 md	101	72	56	0.73	0.11	0.00006	10.493	0.119	0.475	0.005	0.160	0.001	1.01	2504	22	2479	10	2458	6	1.9
M6-5 m	33	37	19	1.15	0.18	0.00010	10.195	0.140	0.460	0.005	0.161	0.001	1.01	2439	24	2453	13	2464	12	-1.0
M6-6 e	27	23	16	0.88	0.47	0.00025	10.534	0.166	0.473	0.006	0.162	0.001	0.97	2496	26	2483	14	2472	15	1.0
M6-7 m	23	23	14	1.01	0.38	0.00021	10.842	0.147	0.486	0.006	0.162	0.001	1.00	2553	25	2510	13	2475	11	3.1
M6-8 m	24	25	15	1.05	0.18	0.00010	10.719	0.159	0.474	0.006	0.164	0.001	1.00	2502	27	2499	14	2496	12	0.2
M6-9 m	69	71	41	1.05	0.15	0.00008	10.576	0.129	0.474	0.005	0.162	0.001	1.00	2503	23	2487	11	2473	9	1.2
M3-1 e	24	24	14	1.03	0.33	0.00018	10.203	0.141	0.461	0.005	0.160	0.001	1.00	2446	23	2453	13	2459	14	-0.5
M3-2 e	48	45	28	0.95	0.20	0.00011	10.304	0.125	0.467	0.005	0.160	0.001	1.00	2470	22	2462	11	2456	9	0.6
M3-3 eb	20	11	10	0.59	0.35	0.00019	10.067	0.152	0.456	0.005	0.160	0.001	0.98	2422	24	2441	14	2456	15	-1.4
M3-4 e	43	33	25	0.78	0.26	0.00014	10.686	0.143	0.478	0.005	0.162	0.001	1.00	2517	23	2496	12	2479	12	1.5
M3-5 md	40	43	23	1.09	0.22	0.00012	10.130	0.124	0.454	0.005	0.162	0.001	1.00	2415	23	2447	11	2473	7	-2.4
M3-6 m	17	13	10	0.75	0.58	0.00031	10.366	0.147	0.467	0.006	0.161	0.001	1.00	2468	26	2468	13	2467	10	0.1
M3-7 eb	28	21	16	0.76	0.29	0.00016	10.244	0.171	0.475	0.006	0.156	0.002	0.99	2504	25	2457	15	2418	19	3.6
M3-8 m	17	14	10	0.83	0.83	0.00045	10.078	0.128	0.468	0.005	0.156	0.001	0.97	2473	23	2442	12	2416	10	2.4
M3-9 eb	30	26	17	0.89	0.20	0.00011	10.871	0.143	0.485	0.006	0.163	0.001	1.02	2550	25	2512	12	2482	8	2.7
M3-10 e	23	23	15	0.98	0.34	0.00018	11.089	0.151	0.498	0.006	0.161	0.001	1.02	2607	24	2531	13	2469	14	5.6
M3-11 mb	22	17	13	0.79	1.29	0.00070	10.780	0.173	0.496	0.006	0.158	0.002	0.99	2597	25	2504	15	2429	19	6.9

*m* megacryst zircon, *e* equant zircon, *d* dark luminescence, *b* bright luminescence, *o* overgrowth, *f*_*206*_ percentage of ^206^Pb that is common Pb, *ρ* error correlation between ^206^Pb/^238^U and ^207^Pb/^235^U ratios; *D* percent discordance from concordia line

### Interpretation

#### Metasomatic origin of veins

The compositions of the vast majority of veins are not representative of liquid compositions as they are solely composed of orthopyroxene. However, their geometry suggests that they form as a consequence of emplacement of melt or fluid. The transition from the evolved leucomonzonite composition of the central part of the largest veins to ultramafic orthopyroxenite at the margins occurs on the scale of a few centimetres (Fig. 3). Hence, the former is unlikely to represent the end product of an extreme fractionation process of an ultramafic parental melt. The mineralogy of the evolved component of the vein is most compatible with crystallization from a Si-rich melt composition. The introduction of such a melt into peridotite, will allow metasomatic interaction between the Si-rich melt and the olivine in the solid-state ultramafic host. The system is dominated by a forsterite breakdown reaction to produce orthopyroxene at the margins of the veins. Similar mineralogical sequences to those observed at Loch an Daimh Mor are recorded by Sekine and Wyllie (1982) in mixing peridotite and granite at 30 kb producing abundant phlogopite and enstatite.

The zoned structure of the veins with low variance mineral assemblages and indeed the scale of this zoning are typical of that generated by metasomatic processes (Arai 1975; Sanford 1982; Barton et al. 1991; Vrijmoed et al. 2013). As such, the leucomonzonite is thought likely to represent the least modified component of the vein material, although the extent of metasomatism is such that the composition of the original melts will be difficult to assess. The larger veins record the most complete sequence of assemblages from more ultramafic compositions to the central more evolved feldspathic compositions. Significant subsolidus transformations are thought to occur within the zoned veins, such as the partial replacement of clinopyroxene by pargasite and the symplectite reaction textures that produce phlogopite in the internal parts of the structure. The overall volumes of melt introduced into the peridotite were probably not especially large, given the scale of the orthopyroxenite veins and the paucity of felsic components. Such volumes are particularly difficult to assess given the uncertainty in the positions of the original margins of the melt conduits, which are masked by the metasomatic transformation of the host peridotite to orthopyroxenite.

#### Origin of the zircon megacrysts

The well-shaped oscillatory zoning around a euhedral core and the similarly euhedral shape of the megacrysts (Fig. 4) indicate that all major stages of the zircon megacryst growth occurred within a fluid or melt phase. As such there is no evidence of any inherited components to the megacrysts. This is supported by the U–Pb age of the megacryst cores being identical to the age of the rims (Table 4). The textural characteristics of the megacrysts are similar to those of kimberlitic zircon (Belousova et al. 1998; Corfu et al. 2003) with both a large size, mosaic texture generated by abundant fractures associated with brittle behaviour, and some multistage but often rather poorly developed zoning. The complex zoning observed in the Loch an Daimh Mor megacryst zircon in our investigation contrasts with the large uniform zircon with no primary growth zoning studied by Timms and Reddy (2009) from the same locality.

The scale of both the zircon megacrysts and equant grains, together with the nature of the growth zoning that is preserved and the consistent geochemical and isotopic signatures of both populations, suggests that they preserve significant elements of their growth chemistry. Although aspects of resetting do occur, as discussed in later sections, these appear to be limited to discrete fractures and some small-scale variations associated with grain edges (Fig. 4). As a result, much of the character of the zircon is independent of the later deformation and alteration events that have modified the CL signatures. Generally, the total REE and Y contents of the megacrysts are higher than those typical of zircon from mantle assemblages (Fig. 6) (Belousova et al. 1998) and low in comparison to zircons from crustal lithologies (Rubatto and Hermann 2007). However, the overall REE patterns are comparable to zircon in some high grade gneisses (Hoskin and Ireland 2000; Whitehouse 2003), including those from mafic gneisses in the immediate vicinity (Fig. 6) (MacDonald et al. 2015). Oxygen isotopic compositions of the non-rim zircon (6.73 ± 0.42‰) are distinct from zircon that is derived from probable mantle-like sources (Fig. 7), which have *δ*^18^O compositions of 5.3 ± 0.3‰ (Valley et al. 1998; Valley 2003). Both the megacrysts and the equant type zircon have *δ*^18^O values similar to those of the Lewisian mafic gneisses (6.4–6.8‰) (Cartwright and Valley 1992) that surround the peridotites (Fig. 7). Such gneisses also have very similar *δ*^18^O compositions to those of the felsic sheets (6.9–7.5‰) that are within the Scourian gneisses nearby (Cartwright and Valley 1992). Given the lack of isotopic fractionation during melting (Taylor and Shephard 1986), these sheets are likely to be derived by anatexis of the host orthogneisses during peak granulite-facies metamorphism (Cartwright and Valley 1992). A small ultrabasic (hornblendite) pod from near Geodh’ nan Sgadan, ca 2 km SW of the present sample site, was also analysed by Cartwright and Valley (1992) and yielded lower *δ*^18^O compositions of 5.5–5.9‰. As such the isotopic composition of the zircon within the peridotite host is most consistent with derivation from the introduction of crustal melts that were probably sourced from the local gneisses.

The age of the zircon megacrysts determined in this study (2,464 ± 12 Ma) is within error of previously determined age of 2,451 ± 14 Ma (Timms et al. 2006) and is interpreted as a crystallization age, broadly similar to the age of a regional high grade metamorphic event of 2,482 ± 6 Ma identified by MacDonald et al. (2015). Bands of felsic melt typically permeate the surrounding mafic gneisses (Johnson et al. 2012) and estimated peak metamorphic conditions in the host gneisses are consistent with the production of partial melts (Johnson and White 2011). Consequently, the U–Pb zircon data are compatible with zircon crystallization during the introduction of crustal melts into the peridotite. The time gap associated with these ages is thought unlikely to be significant. In the absence of evidence for rapid exhumation in this time period, it is probably indicative of the prolonged period of time that such lower crustal lithologies may spend at or close to peak metamorphic conditions. The U–Pb ages are consistent with growth of the zircon megacrysts during a granulite-facies, possibly Inverian event.

The zoning textures and shape of the zircon megacrysts, their “crustal-like” geochemistry, rich in REE and other trace elements are all consistent with the introduction of an evolved Si-rich melt phase of crustal affinity (Boehake et al. 2013) into the peridotite. In addition, the negative Eu anomaly in the zircon megacrysts either indicates the likelihood of contemporaneous plagioclase crystallization (Hoskin et al. 2000) or the melts were depleted in Eu due to plagioclase remaining in the restite of the host gneisses. Zircon megacrysts appear to form as part of an early in situ crystallization sequence associated with orthopyroxene ± phlogopite in the largest of the veins. A broadly crustal origin raises the possibility that zircons are transported into the peridotite as inherited grains with the siliceous melt. Such origins have been inferred in other examples of zircon within ultramafic host rocks (Liu et al. 2009; Belousova et al. 2015; Li et al. 2016), although these are typically not megacrystic. However, there is no indication of inheritance in the megacrysts and the presence of orthopyroxene and phlogopite inclusions in the zircon suggests that they have grown within the veins. The Hf content of the megacrysts is generally compatible with crystallization from a low Si parent melt (Belousova et al. 2002).

The size and local abundance of the zircon must reflect highly mobile components during crystallization and/or locally exceptionally high concentrations of Zr in the liquid. Modelling indicates that the average amphibolite-facies tonalite composition of Rollinson and Windley (1980) would become nepheline normative after removal of around 30% SiO_2_. If SiO_2_ is the only melt component removed during the metasomatism, and the abundance of orthopyroxenite in all vein margins suggests that SiO_2_ is the dominant mobile component in the system, peralkalinity will not change significantly. However, SiO_2_ saturation will change, and miaskitic, nepheline (and potentially, leucite) normative melts may be produced. By itself this process will only produce minor enrichment of Zr in the remaining Si-undersaturated melt phase. However, it will dramatically change the behaviour of Zr in the melt, by increasing zircon solubility, suppressing zircon nucleation, promoting depolymerization of the melt (Ni et al. 2015) and allowing melt Zr levels to build up in residual melts/fluids, such that the megacrysts may form (cf. Schaltegger et al. 2015).

Significant mobility of Zr within the melt is required to allow the growth of megacrysts and this mobility seems to persist as shown by the late stage crystallization of interstitial zircon in the phlogopite-rich zone (Fig. 3e). Such geochemical mobility is generally compatible with the low variance assemblages in the zoned veins. Scapolite within metasomatic assemblages is commonly reported from other studies to be associated with late replacement of plagioclase in the presence of a volatile-rich fluid (Ekström 1972; Drivenes et al. 2016). S-rich scapolite as found in these assemblages is restricted to granulite-facies rocks (e.g. Lovering and White 1964; Hammerli et al. 2017). The presence of phlogopite in the vein assemblages and the ubiquitous fracturing of zircon grains may also point towards elevated volatile contents. High volatile contents of the melts will enhance HFSE solubility irrespective of melt composition (Antignano and Manning 2008; Rapp et al. 2010; Wilke et al. 2012; Louvel et al. 2014).

Initial crystallization of orthopyroxene occurs before the liquid is saturated in Zr. However, the bulk of zircon is associated with the orthopyroxenite rather than the more evolved fractions of the veins, suggesting that the incoming melt rapidly approaches zircon saturation following initial desilicification. A build up of Zr in the residual melt might be expected to be linked to an increased concentration of other HFSE. This is not observed in any of the veins, which lack abundant apatite or prominent Ti-bearing minerals, although potentially phlogopite may incorporate some of the likely residual components.

### Clustering of megacrystic zircon within the veins

Overall, the abundance of zircon may not be exceptional, because most of the veins lack zircon. However, locally extreme Zr contents are recorded (Fig. 2e) and potentially could be caused by three mechanisms:

1. Local emplacement of exceptionally Zr-rich melts

The adjacent TTG gneisses contain textural evidence of partial melting (Johnson et al. 2012) and record granulite-facies metamorphism that post-dates the formation of the peridotites and so provide a potential and proximal source of felsic melts. Melts from Lewisian granulite and amphibolite-facies rocks have average Zr contents between 100 and 150 ppm (Rollinson and Windley 1980), although a large component of this is probably inherited restitic zircon. Metaluminous to peraluminous granitic melts with over 50–100 ppm Zr will be saturated with zircon (Watson 1979). Consequently, it is difficult to produce melts with higher levels of Zr by anatexis. If Zr contents of the melts were 50 ppm, 100 kg of melt would contain enough Zr to form 10 g of zircon or roughly 0.01% zircon. Hence, the zircon concentrations in these orthopyroxenite veins are locally more than two or three orders of magnitude greater than would be predicted from a “typical” crustal melt. This coupled with the absence of textural evidence for the emplacement of multiple melts of different compositions argues against the introduction of unusual Zr-rich melts.

2. Clustered nucleation of zircon

Reaction with the peridotite wall rocks and desilicification of the felsic melts will ultimately cause those melts to reach zircon saturation. These processes may be locally enhanced by the geometry of the vein systems and the concentration of residual melt. Although the 3D geometry of the network of the veins is hard to assess, zircon appears to be preferentially concentrated in some of the larger veins. Hence, a factor associated with original vein geometry and or width seems most likely to control the clustering of the zircon. Once nucleation has occurred in these veins, Zr is delivered to these clustered crystals within the remaining melt network in the peridotite. The continued growth will allow the formation of the megacryst clusters. Such localized nucleation and subsequent growth may be characteristic of the other vein components in this metasomatic environment, such as the localized lenses of clinopyroxene.

3. Clustering of zircon by flow sorting

A physical accumulation of zircon could occur through either density settling or more probably, given the scale of the vein system, via flow sorting. The complex geometry of the veins, the large size of the megacrysts relative to the scale of the veins and the likely crystallinity of the veins at the time of zircon crystallization all apparently mitigate against physical separation of zircon due to the perceived difficulty in maintaining active sustained flow paths. The lack of disturbance to the growth zoning suggests that megacrysts would need to be transported intact within a relatively permeable melt network in which flow is maintained for a prolonged period. They may then preferentially accumulate in parts of the channel network where flow rates are lowered. Crystallization of these veins may be slow, associated with the ambient granulite-facies conditions recorded in the gneisses and the evidence of slow cooling from the coarse grain size of the vein assemblage. However, the occurrence of zircon within a low variance assemblage in isolation of other phases and the lack of alignment suggests that the clustering of megacrystic zircon by flow sorting is unlikely.

### Subsolidus behaviour of zircon

A later stage of zircon growth and recrystallization follows in subsolidus conditions and is represented by clusters of small equant granoblastic zircon around the edge of many megacrysts (Fig. 5). This recrystallization process appears to match that of the co-existing orthopyroxene (Fig. 3a) with the transformation of megacrystic morphologies into granoblastic grains. The zoning and textural characteristics indicate that the equant zircon partly represents a replacement phase (i.e. growth towards the megacrysts) and partly nucleation and growth of new grains in clusters immediately adjacent to the megacrysts. This group of zircon has more metamorphic (solid-state) characteristics with broader zoning and granoblastic textures. The presence of thin bridges of bright luminescent zircon between the two types (Fig. 5d) does indicate that minor growth of the megacrysts may locally continue after the growth of the granular zircon. The 2465 ± 12 Ma U–Pb age of the equant zircon is within error of the age of the megacrysts and hence this recrystallization event must occur during the same broad period of activity, and given the uncertainty in the age determinations within a maximum ca. 23 Ma of the megacryst growth.

The nature of the zircon generated by both recrystallization processes and new growth is distinctive and contrasts with the more irregular-shaped patchy recrystallization that characterizes most low temperature alteration of zircon (Hay and Dempster 2009). Even examples of higher temperature recrystallization reactions (Hermann et al. 2006; Liati and Gebauer 2009) tend to not produce the equant granoblastic zircon associated with the replacement of megacrysts in the orthopyroxenite veins. This may be indicative of a combination of high temperatures and/or long time scales associated with this process.

The later generation of zircon is trace element poor and has a REE chemistry that may be more representative of the peridotite/orthopyroxenite and the less pronounced Eu anomaly may be indicative of no contemporaneous plagioclase crystallization. The relative depletion of REE in this population could also be indicative of crystallization of amphibole and phlogopite in the vein system. In comparison to zircon from kimberlites, HREE content of the equant zircon are enriched, probably reflecting the absence of co-existing garnet (Rubatto and Hermann 2007). However, generally trace element contents of this population are typical of zircon with mantle-like affinities (Kresten et al. 1975; Belousova et al. 1998; Hoskin and Schaltegger 2003; Robles-Cruz et al. 2012), although the LREE content is higher in the zircon from this study. In part, this may reflect the combination of geochemical inheritance from the adjacent megacryst, an increased influence of the ultramafic host and a reduced influence of the crustal melt that formed the original vein. The equant zircon population reveals evidence of greater heterogeneity of oxygen isotopic compositions than the megacrysts perhaps reflecting crystallization within a less open geochemical environment or more variable later conditions. The outer edge of the equant zircon in contact with the megacryst may have a transitional chemistry with a HREE composition similar to that of the adjacent megacryst and a LREE content similar to that of the rest of the equant grains (Fig. 6). This implies a difference in the mobility of the REE during the recrystallization process, with enhanced mobility of the LREE.

The recrystallization apparently post-dated some of the fracturing that affected the megacrysts, although the megacrysts may be more prone to strain and an inability to rotate. As such determining a history of deformation events solely based on such textural criteria may be difficult. Such deformation events may have enhanced new nucleation in the progressively more crystalline system. A variety of events (e.g. Inverian and Laxfordian) potentially affect both generations of zircon and both zircon growth and chemical modification of the actinide content of zircon may be associated with these events. The actinides appear to be the most mobile of the trace elements within zircon (Timms et al. 2006). The lack of any correlation between REE content and luminescence brightness suggests that rather than a REE-activated CL response (Timms and Reddy 2009), the luminescence in these grains may be more a function of radiation damage. However, the lack of difference in the U–Pb isotopic ages suggests that most of this chemical modification occurred shortly after the growth of the zircon and that high temperature conditions persist during brittle deformation of the zircon. Later diffusive modification along grain boundaries occurs associated with the bright luminescent rims to all grains and is associated with a late stage fracturing and probably fluid ingress along fractures and grain boundaries. Some of the bright rims are “balanced” by an immediately adjacent dark luminescent zircon so small-scale chemical redistribution of actinides occurs. These have an apparently lower ^207^Pb/^206^Pb age of ca. 2,418 ± 19 Ma and are possibly related to the time of Scourie dyke emplacement. The slightly higher δ^18^O values that appear to characterize the rim compositions might also be broadly consistent with trends observed within the retrograde Inverian events within the host gneisses (Harmon 1983; Cartwright and Valley 1992). Fracturing is also localized around grain edges with a variety of fracture morphologies. Curved fractures centred around a point on the zircon surface, are interpreted to result from the impact of adjacent strong minerals acting as point sources. The last obvious phase of deformation is associated with large fractures that are serpentine-filled with up to mm-scale displacement with fragments of zircon within the serpentine.

## Discussion

The presence of zircon in ultramafic assemblages is reported from a number of different environments (Kresten et al. 1975; Rubatto and Hermann 2003; Hermann et al. 2006; Page et al. 2007; Marrochi et al. 2009; Yang et al. 2016), however, it is typically associated with crystallization in silicic melts of broadly crustal affinity (Hoskin and Schaltegger 2003). As such its potential role in ultramafic rocks is perhaps not widely appreciated. The pyroxenite veins at Loch an Daimh Mor are associated with metasomatic reaction between crustal-derived Si-rich melts and a peridotite host, resulting in melt desilicification and suppression of zircon saturation, and enhanced Zr mobility within the Si-undersaturated melts. In these high temperature conditions, melt networks remain open for an extended time to allow for the physical concentration, or prolonged growth of zircon megacrysts in parts of the vein network. The textures and geochemistry of the megacrysts bear comparison with others reported from ultramafic hosts (Belousova et al. 1998; Marocchi et al. 2009) and suggest similar origins.

Zircon may potentially have a variety of modes of formation within the mantle (Valley et al. 1998; Liu et al. 2009; Belousova et al. 2015) but studies typically infer an element of metasomatism with a wide variety of proposed metasomatic melts/fluids (Dawson et al. 2001; Mirnejad and Bell 2006). Those from kimberlites all appear to form in similar metasomatic environments and display a continuum of geochemical characteristics (Page et al. 2007). Zircon from the Loch an Daimh Mor peridotites is texturally and geochemically similar to those of mantle xenolith zircon sampled by kimberlites with generally elevated REE contents (Hoskin and Ireland 2000; Konzett et al. 2000) but the latter lack the prominent Eu anomaly due to the absence of plagioclase in the deep mantle. The equant zircon has a REE geochemistry more similar to those from relatively depleted mantle sources (Robles-Cruz et al. 2012), although with an enriched HREE content. Ti levels are also generally compatible with those from a mantle origin (Page et al. 2007). Mosaic-like textures with fracture-related domains within the zircon megacrysts appear to be similar to those from kimberlite zircon, which are also typically megacrystic (Corfu et al. 2003). This may imply a similar mode of formation and reflect the relative rheology of peridotite host and zircon.

Metasomatic mineral assemblages similar to those in the Lewisian complex are reported from various Alpine peridotites (Zanetti et al. 1999; Markl et al. 2003; Scambelluri et al. 2006; Liati and Gebauer 2009) with interaction between the ultramafic rocks and crustal melts. In a similar context, Marocchi et al. (2009) document zircon megacrysts associated with reactions between peridotite and adjacent crustal gneisses. Metasomatic interactions of this type should in theory be common at subduction zones with crustal slab-derived components infiltrating the mantle wedge from either partial melts or fluids derived from of the slab itself (Prouteau et al. 2001) or from the introduction of subducted sediments (Currie et al. 2007). Zircon, and/or strongly Zr-enriched melts may be produced in such interactions and should be considered with respect to geochemical budgets (Sorensen and Grossman 1993). Crystallization of zircon will deplete melts in HFSE such as Zr and Hf (cf. Kelemen et al. 1990; Thirlwall et al. 1994) and such depletions have been linked to a range of models explaining the geochemistry of arc magmas (e.g. Stern and Kilian 1996; Peate et al. 1997; Münker et al. 2004). In this respect, our findings may have significance in demonstrating the stability of zircon in metasomatised ultramafic rocks (Louvel et al. 2013).

Second, our results have significance to the scales of geochemical heterogeneity and mobility during metasomatic processes (Louvel et al. 2013). The localized clustering of dense megacrystic zircon within narrow vein systems generates significant heterogeneity of HFSE distribution within the ultramafic host. This is notable, given the likely introduction of just a single pulse of melt, but also emphasizes the probable longevity of such melt systems at depth. This is complemented by the enhanced geochemical mobility in the Si-undersaturated melts but contrasts with the limited geochemical mobility in the sub-solidus state. Thus, oxygen isotopic ratios record a transition between homogeneous open system behaviour and heterogeneous compositions in the equant zircon as the melt channels progressively crystallize. In contrast, REE concentrations are largely decoupled from the HFSE, although the LREE show evidence of slightly more enhanced mobility than the HREE during these subsolidus processes. Such behaviour confirms decoupling of HFSE from REE (Rubatto and Hermann 2003) and decoupling of LIL from HFSE during metasomatism (Griffin et al. 1996).

Third, despite the likely prolonged and high temperature history of the zircon, the original trace element signatures of the zircon remain largely intact during subsequent alteration and deformation. Ironically, given the reputation of geochemical robustness of zircon is built around the preservation of old U–Pb ages (e.g. Wilde et al. 2001), it is only the actinides that appear to have been disturbed by the later deformation events (Timms et al. 2006; Piazolo et al. 2016).
